# Influence of Diabetes during Pregnancy on Human Milk Composition

**DOI:** 10.3390/nu12010185

**Published:** 2020-01-09

**Authors:** Chiara Peila, Diego Gazzolo, Enrico Bertino, Francesco Cresi, Alessandra Coscia

**Affiliations:** 1 Complex Structure Neonatology Unit, Department of Public Health and Paediatric, University of Turin, 10100 Turin, Italy; enrico.bertino@unito.it (E.B.); francesco.cresi@unito.it (F.C.); alessandra.coscia@unito.it (A.C.); 2 Department of Maternal, Fetal and Neonatal Health, C. Arrigo Children’s Hospital, 15121 Alessandria, Italy; dgazzolo@hotmail.com

**Keywords:** human milk, breastfeeding, gestational diabetes mellitus, insulin-dependent diabetes mellitus, preterm newborn

## Abstract

Human milk (HM) is a unique nourishment believed to contain biological factors contributing to both short and long-term benefits. Considering that a mother’s own milk is often considered the first choice for nutrition of neonates, an aspect of increased interest is the possible effect of diabetes on the mammary gland and therefore on breast milk composition. This article aims to review the published literature on this topic, and to offer additional insights on the role of this disease on the composition of HM. This review was performed by searching the MEDLINE, EMBASE, CINHAL and Cochrane Library databases. A total of 50 articles were selected, focused specifically on one of the two types of diabetes: gestational diabetes mellitus (21 studies) and insulin-dependent diabetes mellitus (8 studies). Overall, the findings from the literature suggest that diabetes can alter the composition of HM. Nevertheless, the studies in this field are scarce, and the related protocols present some limitations, e.g., evaluating the variability of just a few specific milk biochemical markers in association with this syndrome.

## 1. Introduction

Human milk (HM) is a specie-specific biological dynamic fluid and significantly varies from one woman to another. HM constantly changes during lactation to adapt to the physiological needs of the developing infant [1] and this also comes about because of the gradual maturation of the mammary gland, which depends on the placenta and thus on the progress of the pregnancy. The policy statement of the American Academy of Pediatrics (AAP) regarding breastfeeding affirms that “Breastfeeding and human milk are the normative standards for infant feeding and nutrition” [1]. In addition, the AAP highlights that the mother’s own milk, fortified appropriately, should be the primary diet also for the preterm newborns. These recommendations are due also to the fact that breastfeeding has been shown to have beneficial effects on short- and long-term maternal and infant health outcomes [1,2,3]. 

It has been proposed that nutrition signals during the early postnatal period may influence metabolic developmental pathways and induce permanent changes to metabolic disease susceptibility [4,5]. In support of these hypotheses, studies have reported that human milk (HM) has a protective effect on obesity and type 2 diabetes later in life [5,6]. Benefits of HM are mediated by specific bioactive substances that transiently regulate tissue activities while the neonate’s systems mature [7,8]. Biological active components, such as hormones, immunoglobulins, lysozyme, lactoferrin, saccharides, nucleotides growth factors and enzymes antioxidants, are involved in immunological and metabolic regulation, and it has been hypothesized that they mediate growth and development in infancy [9,10]. Furthermore, HM is a “dynamic” system: the composition changes and is influenced by several conditions, such as term-preterm delivery, maternal diet, metabolic abnormalities and pathologies [11]. 

Diabetes is a pathological condition of the lactating mother that may preexist (type 2 diabetes, type 1 diabetes or insulin-dependent diabetes mellitus (IDDM)) or appear during the pregnancy. The latter case is identified as gestational diabetes mellitus (GDM) and is a common pregnancy complication. It is defined as a carbohydrate intolerance of variable severity, with onset or first recognition during pregnancy. Diabetes is associated with several short- and long-term complications for the mother and the newborns and it is known that this pathology can delay the onset of lactogenesis II and affect the composition of the human milk [12]. Bearing in mind the important impact of the HM on the development of the newborns and the potential effects of the diabetes on the composition of the milk, several studies were conducted to evaluate the variability of milk biochemical markers in association with this pathology. 

A comprehensive review and comparison of the related data on the effects of diabetes conditions on the specific composition of HM of the lactating mother is, to the best of the authors’ knowledge, not available in the literature. Our aim is to assess the current knowledge on the interactions between diabetes in pregnancy and the composition of the HM in order to better understand the potential effects of this pathology on the nutrition and development of newborns. 

## 2. Materials and Methods 

The literature review was performed by conducting electronic searches of MEDLINE (via PubMed and PubMed Central), EMBASE, CINHAL and the Cochrane Library. The electronic search used the following keywords and MeSH terms: (i) human milk AND (gestational diabetes OR gestational diabetes mellitus OR insulin-dependent diabetes mellitus OR type 2 diabetes); (ii) breastfeeding composition AND (gestational diabetes OR gestational diabetes mellitus OR insulin-dependent diabetes mellitus OR type 2 diabetes); (iii) breast milk AND (gestational diabetes OR gestational diabetes mellitus OR insulin-dependent diabetes mellitus OR type 2 diabetes); (iv) preterm breast milk AND (gestational diabetes OR gestational diabetes mellitus OR insulin-dependent diabetes mellitus OR type 2 diabetes); (v) human milk composition AND (gestational diabetes OR gestational diabetes mellitus OR insulin-dependent diabetes mellitus OR type 2 diabetes); (vi) breast milk composition AND (gestational diabetes OR gestational diabetes mellitus OR insulin-dependent diabetes mellitus OR type 2 diabetes). 

No publication date limits were set. For a complete comprehension of the studies, the inclusion criteria were: (i) primary (original) research published in a peer-reviewed journal in the English language and (ii) full text available. For the same reason, case reports, commentaries, letters to the editor, and reviews were excluded. Articles including data related to animal milk were also excluded. 

Literature searches were performed in the period between 1 June 2019 and 1 November 2019.

## 3. Results

**Figure 1:**
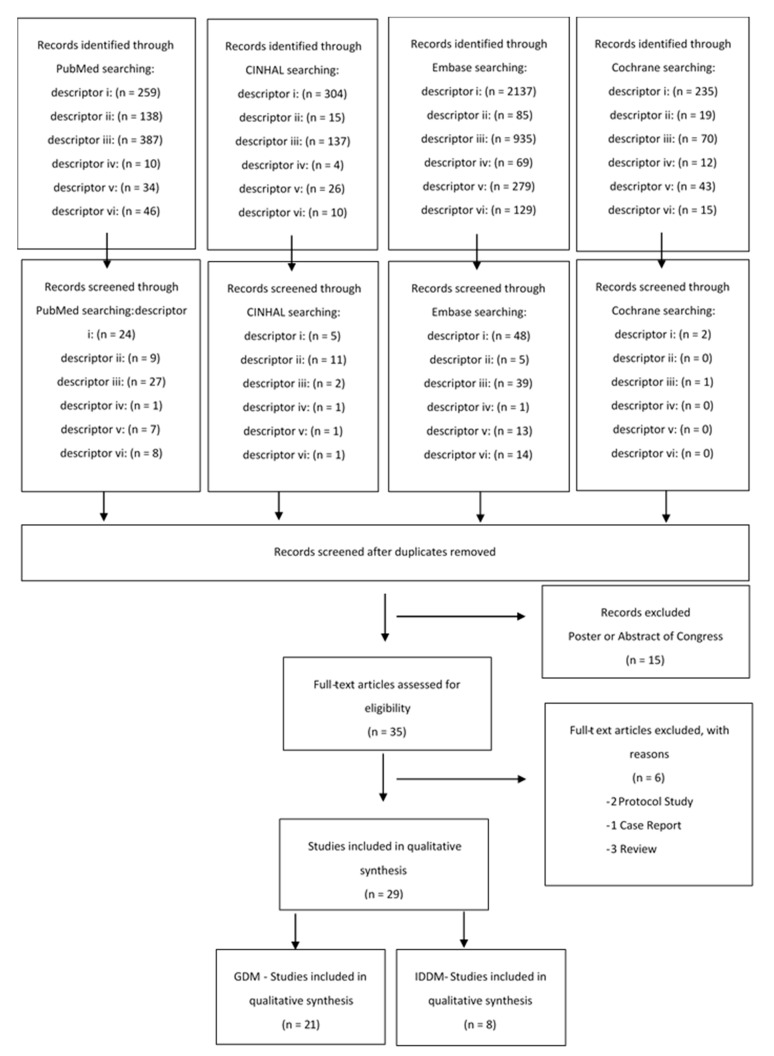
Flow diagram of the different phases of the review.

A total of 50 articles were found from a combination of the searches, but only 29 fulfilled the inclusion criteria, as shown in the flow diagram below, Figure 1. 

The selected literature studies were focused specifically on one of two types of diabetes: GDM or IDDM. In line with this finding, we evaluated the studies separately, based on the diabetes type. Since type 2 diabetes was only referenced in a single article and compared to GDM, it is not discussed separately in our review. 

The available literature data on GDM and HM are summarized in Table 1 and Table 2, whereas data on IDDM and HM are summarized in Table 3 and Table 4.

### 3.1. Gestational Diabetes Mellitus—GDM

We found 21 articles focused on gestational diabetes mellitus; the related information is summarized in Table 1 and Table 2.

#### 3.1.1. Energy

The energy content of the HM of women diagnosed with GDM was evaluated in two studies, with opposite results: one study showed higher energy content in all phases of lactation with respect to healthy mothers, whereas the other study reported a lower energy content in the mature milk of pathological mothers. Both studies used an MIRIS scanner for the analyses of the samples; the differences only consisted of the methods used to collect and store HM samples [13,14].

#### 3.1.2. Protein Content

The total protein content was investigated in three studies, with similar results concerning the colostrum: no significant differences were found with respect to the control group. Nonetheless, data reported in relation to transitional and mature milk were conflicting: Dritsaku et al. found a reduction in concentration of both types of HM, whereas the results of Saphira et al. showed no differences [13,14,15].

#### 3.1.3. Hormones

Insulin was evaluated in several studies, both in colostrum and mature milk. Ley et al. used an electrochemiluminescence immunoassay and showed that insulin concentrations in early milk were higher than those in mature milk. They found that maternal prenatal metabolic measures, including higher pre-gravid body mass index (BMI), gravid hyperglycemia, insulin resistance, lower insulin sensitivity and higher serum adiponectin were associated with higher insulin concentrations in mature milk, whereas these factors were not associated with altered insulin concentrations in early milk [16]. Another two studies analyzed the HM samples with an ELISA test, but found opposite results. Yu et al. showed an increase of insulin levels, not only in mature milk, but also in colostrum in women with GDM, especially in those receiving insulin injections [17]. Nunes et al. did not find differences in both types of milk [18].

Adiponectin levels showed similar conflicting results: two studies demonstrated that GDM was not associated with variations of adiponectin levels in HM, whereas Yu et al. observed that women with GDM had lower concentrations of adiponectin in colostrum on day 3 and mature milk on day 90, but no differences on day 42 [16,17,18].

There is agreement in the Ghrelin data: Ghrelin levels were found to be lower in colostrum and mature milk, but no differences were observed in transitional milk [17,19,20].

Irisin concentrations, analyzed in two studies, were accordingly found to be low in GDM subjects in the colostrum and transitional milk [21,22]. In mature milk, Fatima et al. observed a reduction in concentrations, not confirmed by Aydin et al. [21,22]. In addition, the study by Aydin et al. found Irisin levels to increase from the colostrum to transitional and mature milk in both normal glucose tolerant and GDM patients [22].

Several other biologically active components with hormonal activity are evaluated in one study by the same group of researchers. The study considered only mothers who did not take insulin, with no complications during pregnancy and who had full term deliveries. Instead, the concentrations of Apelins and Nesfatin-1 in HM in GDM lactating women was lower than in the control samples and the concentrations of Apelins and Nesfatin-1 were higher in the mature milk than in colostrum [19]. Copeptin, adropin and polypeptide are hormones implicated in energy homeostasis and diabetes [22]. The authors found that the copeptin concentration was significantly higher in colostrum than transitional milk, and the highest concentration was in mature milk from women with or without GDM [22]. Conversely, the adropin concentrations were significantly lower in the colostrum than in transitional milk, and the lowest concentration was in mature milk from women with or without GDM [22]. Preptin, salusin-alpha and -beta and pro-hepcidin and hepcidin-25 concentrations were evaluated in all phases of breastfeeding [23]. The data demonstrated that women with GDM had significantly higher colostrum preptin and colostrum/transitional pro-hepcidin and hepcidin-25 concentrations than healthy lactating women. Salusin-alpha and -beta levels were lower in colostrum of women with GDM [23]. SREBP1-c profile showed a level reduced from a very low value to an undetectable range in colostrum and mature breast milk, respectively [21].

#### 3.1.4. Anti-Infective Proteins

IgA content was evaluated in the colostrum in one study and in transitional milk in another study [15,24]. No differences were observed in the colostrum, while a decrease of total protein and glycosylation of sIgA was found in transitional milk. Lactoferrin was analyzed only by Smilowitz et al. in transitional milk, observing an increased glycosylation of this protein. The results suggest that maternal glucose dysregulation during pregnancy has lasting consequences that may influence the innate immune protective functions of HM [24].

Ustebay et al. analyzed Chemerin and Dermicin in all phases of lactation and found that these are significantly increased by GDM; moreover, the highest amount was found in the colostrum and the lowest in mature milk [25].

Metallinou et al. evaluated the levels of Neutrophil gelatinase-associated lipocalin (NGAL) and its complex with matrix metalloproteinase-9 (MMP-9) in the colostrum and found that the mean complex concentration was significantly higher in diabetic pregnancies in comparison to normal ones [26].

#### 3.1.5. Proteomics Profile

The proteomics profile was assessed in two studies, using different techniques (high-performance liquid chromatography or high-sensitivity, label-free, semiquantitative mass spectrometry) [27,28]. Klein et al. evaluated the content of 11 free amino acids (FAAs) in the colostrum and mature milk in women with or without GDM. The authors found that the total amount of FAA increased from colostrum to mature milk in all patients. Moreover, the total amount did not significantly differ between groups, as well as the concentrations of each individual amino acid [27].

Grapov et al. determined the proteomic profiling only of colostrum. A total of 601 proteins were identified, of which 260 were quantified. Orthogonal partial least-squares discriminant analysis identified 27 proteins that best predict GDM. The power law global error model, corrected for multiple testing, was used to confirm that 10 of the 27 proteins were also statistically significantly different between women with GDM and those without GDM. The identified changes in protein expression suggest that diabetes mellitus during pregnancy has consequences on human colostral proteins involved in immunity and nutrition [28].

#### 3.1.6. Saccharides

The total saccharides content was evaluated in two studies. Both found no differences between groups in the colostrum and transitional milk, although different results regarding mature milk were reported: Saphira et al. observed a lower concentration in GDM, while Dritsaku et al. did not find differences [13,14].

Lactose and glucose were analyzed only in one study and only in the colostrum; lactose was found to be lower in GDM women, whereas glucose seemed unaltered by this pathological condition [15].

In conclusion, a single study evaluated the oligosaccharides in transitional milk and no differences between groups were found in the results [24].

#### 3.1.7. Lipids

The total lipid content was evaluated in three studies with discordant results. Kaushik et al. analyzed only the colostrum and found a reduction in total content in the GDM group [15]. On the other hand, Dritsaku et al. and Saphira et al. did not show any difference in concentration. Similarly, regarding mature milk, Saphira et al. found a reduction, but Dritsaku et al. did not observe a significant difference. Regarding transitional milk, all studies agreed and found no differences between groups [13,14].

Kaushik et al. also analyzed the concentration of triglycerides and cholesterols in colostrum; in this case no differences in content in GDM HM were identified [15].

Azulay Chertok et al. examined the effect of GDM on colostrum fatty acid composition. Analyses of the fatty acid composition revealed significantly higher concentrations of four essential ω-6 polyunsaturated fatty acids (γ-linolenic, eicosatrienoic, arachidonic, and docosatetraenoic) in the colostrum of GDM women, as compared to non-GDM women [29].

#### 3.1.8. Electrolytes

Sodium was evaluated in two studies in colostrum, and both showed an increase of sodium concentrations in correlation to GDM; moreover Galipau et al. found an increase in relation to insulin use [15,30]. Potassium, phosphorus and calcium were analyzed by Kaushik et al. in the colostrum and no differences were found [15].

#### 3.1.9. Vitamin E

GDM was not associated with changes in α-tocopherol concentration in the colostrum. No correlation was found between the concentration of α-tocopherol in the serum and in the colostrum for control and diabetic groups [31].

#### 3.1.10. MicroRNA

A recent study evaluated the microRNAs levels (let-7a, miRNA-30B and miRNA-378) in HM, all of which are known to participate in adipogenesis. Data showed a significant difference in let-7a and miRNA-378 among the normal group and the GDM group. However, these differences disappeared by controlling maternal pre-pregnancy BMI, because pre-pregnancy BMI was a confounder that was correlated to gestational metabolic complications [32].

#### 3.1.11. Metabolome

Wen et al. determined the human milk metabolome profile of GDM women over the first month of lactation. A total of 187 metabolites were identified in the breast milk, including 4 alkanes, 17 amino acid derivatives, 21 amino acids, 22 saturated fatty acids, 29 unsaturated fatty acids, 8 TCA cycle intermediates, 3 cofactors or vitamins, 3 keto acids and derivatives, 1 glycolytic intermediate, 43 organic acids, and 36 organic compounds. The metabolome composition and differences among the colostrum, transition milk, and mature milk from GDM mothers shared many similarities with those from normal pregnancies. However, there were 28 metabolites that were found to be significantly different between women with normal pregnancies and women with GDM pregnancies in the colostrum, transition milk, and mature milk samples [33].

### 3.2. Insulin-Dependent Diabetes Mellitus—IDDM

We found eight studies that focused on IDDM and all were published before the 21st century. These articles evaluated the HM of diabetic women with diagnosis and exordium before the pregnancy, with different durations of the pathology and different controls of the therapies. Four studies were conducted based on the same protocol.

The results are summarized and reported in Table 3 and Table 4.

#### 3.2.1. Proteins

Three studies did not find a difference in concentrations in total protein content between groups, although several differences were found in the protocol, as shown in Table 3 [34,35,36].

Only one study evaluated the lactoferrin and secretory IgA and no differences were found between IDDM mothers and healthy mothers [34].

Another study investigated prolactin levels and found that, in the first postnatal week, milk immunoreactive prolactin concentrations were lower for women with IDDM than for the control group [37]. The significantly lower milk prolactin concentration could be related to elevated serum glucose. High postprandial capillary glucose explained much of the variance in HM prolactin measurements for IDDM mothers. The good glycemic control during pregnancy and early postpartum period was associated with higher perinatal milk prolactin values. Moreover, the inverse relationship between lactose and milk prolactin, which was significant at day 2 postpartum for reference women, was delayed until day 14 postpartum for women with IDDM. These data reflect the delay in lactogenesis and in establishment of lactation experience by IDDM mothers [37].

#### 3.2.2. Lipids

Lipids are the nutrients more extensively studied in the relationship between IDDM and HM composition: we found four different studies on this topic. The results are quite discordant: three found no changes in total fat, but the data from Bitman et al. had a mean fat content 1.5 times lower in IDDM [34,35,36,37].

The cholesterol level was tested in two studies, with opposite results: one observed five times lower concentration, while the other observed no differences [35,36].

The triglycerides were evaluated in one study and no differences were found [36].

Fatty acid profile was analyzed in three different studies [35,36,37]. Van Beusekom et al. did not find abnormalities in total fatty acid composition [36]. Bitman et al. demonstrated a decreased medium-chain fatty acid, suggesting impairment of fatty acid synthesis in the mammary gland, and an increased oleic acid and high concentrations of polyunsaturated fatty acids, suggesting increased chain elongation [35]. Ferris et al. reported that medium-chain fatty acids in the group with IDDM were similar to or greater than those of the control and reference groups at all times, and were within normal reported ranges. In addition, HM long-chain polyunsaturated fatty acids were lower in women with IDDM from 14 to 84 days postpartum [38].

#### 3.2.3. Carbohydrates

Three studies evaluated the carbohydrate levels, per se, such as the macronutrients of HM [34,35,36]. The data agree in terms of lactose levels and no differences were found [34,35,36]. Results are discordant for the glucose levels: Butte et al. observed that milk glucose was 2.3 times higher in IDDM mothers, whereas Van Beusekom et al. did not find differences [34,36].

In the end, one study analyzed the myoinositol levels and no differences were found [36].

#### 3.2.4. Electrolytes

There were no differences in HM levels of potassium, calcium, magnesium between groups in two different studies. The results of these studies are discordant regarding sodium: Butte et al. reported that sodium was 1.2 times higher, but Bitman et al. did not find differences [34,35].

Moreover, no differences were found between groups for phosphorus, zinc, copper, iron [34] concentrations in one study, and chlorite and citrate levels in another study [35].

#### 3.2.5. Vitamins

Only one vitamin (i.e., alpha tocopherol) was analyzed in one study in IDDM HM. Tocopherol decreased by 50% in all groups between 7–14 days postpartum [39], with no difference between groups.

#### 3.2.6. Markers of Lactogenesis

Neubauer et al. and Arthur et al. evaluated the markers for the onset of milk secretion [40,41]. Arthur et al. reported that the peak in milk lactose occurred significantly later for women with IDDM, suggesting that lactation was delayed. Concentrations of lactose, citrate and glucose increase significantly in milk of IDDM between day 2 and 3. The plateau was reached for all metabolites on day 4 [37]. These data confirm the results by Neubauer et al., finding a significantly lower lactose and higher total nitrogen in the colostrum. Milk lactose increased significantly over time to 42 days postpartum. Breast milk lactose was inversely correlated with breast milk conductivity at 3 days postpartum. Total nitrogen was also inversely correlated with milk lactose for women with IDDM. Conductivity and osmolality did not differ among the three group [40].

## 4. Discussion

Diabetes is a frequent pathology during pregnancy that potentially determines several complications and is a significant risk factor for newborn morbidity [6,42]. The impact of maternal metabolic abnormalities on early postnatal nutrition and infant metabolic curves is of considerable interest, because the offspring of women, both with preexistent diabetes or GDM, are at increased long-term risk for type 2 diabetes, although epidemiologic evidence has also shown a long-term protective effect of breastfeeding against obesity and type 2 diabetes in offspring [1,2,3,43]. Based on this strong correlation between maternal and neonatal morbidity, it is important to know the effects of this condition on the composition of HM, especially in relation to the benefits mediated by the HM for newborns [1,2,3]. In the absence of any clear conclusion about this topic, clinical practice should continue to promote and support breastfeeding, as indicated in recognized international guidelines [1], also for GDM women.

Until now, few studies have been reported in the literature assessing the composition of the human milk of diabetic mothers. The main observation is that GDM and IDDM can alter the composition of HM milk. The effect on HM is present not only in the first day post-partum, but is continued throughout all of the lactating phases. These studies hypothesize that the modification inducted by GDM have a potential role that can be, not only detrimental but also protective for the babies. The data on IDDM highlight that the modification of the composition is related to the control of the pathology.

However, we believe that these studies present some limitations:

(i) Several studies analyzed only one phase of milk and did not consider the variation at different time points during lactation. In addition, the definition of the different phases of lactation are not univocal, i.e., the time of sampling of colostrum ranges between 24 h and 7 days postpartum.
(ii) Some studies do not have a case/control protocol focused on GDM or IDDM, but rather, evaluate the effect of preterm delivery; diabetes is considered in metanalysis as a risk factor.
(iii) Concomitant drug therapies are rarely considered and analyzed.
(iv) The studies focused on IDDM are all published before 21st century, and four have the same protocol study.
(v) No dedicated study focused on type 2 diabetes is present in literature.

## 5. Conclusions

The number of available studies on this topic is scarce and they evaluate the effects of diabetes on a few specific milk biochemical markers with several and heterogeneous variables. Bearing in mind the previous considerations, it is therefore difficult to quantify the real effect of this pathology on the different components of HM, or on the health benefits mediated by mother’s milk for the child. Future studies are needed to obtain a more comprehensive evaluation. Moreover, we believe that the protocols should be assessed with case/control structure, specifically focused on preterm delivery and with a match on gestational age (GA). It will also be important to analyze the effect of different diabetes drugs on HM and their potential interaction on the different biological components. Finally, considering the rising prevalence of type 2 diabetes in association with obesity, we believe it is important to plan future studies to determine possible effects on human milk in this population. These future findings are important to individualize and modify the maternal therapies and supplement the nutrition of the preterm newborns.

## Figures and Tables

**Table 1:** Materials and methods of the different studies included in the survey regarding gestational diabetes mellitus (GDM) and human milk (HM). (Col: colostrum; Trans: Transitional milk; Mat: Mature milk).

Study	GDM Treatments	Sample Time	Gestational Age	Parameters Analyzed	Sample Size (GDM vs. Control Women)
[13]	Diet	Col: 0–5 days Trans: 6–14 days Mat: >14 days	Preterm	Macronutrients and energy	27 vs. 183
[14]	Diet Other medications	Col: 72 h Trans: 7 days Mat: 14 days	Term	Macronutrients and energy	31 vs. 31
[15]	Not evaluated	Col: 2–4 days	Term	Total proteins Total lipids Triglycerides cholesterol Lactose Glucose Calcium Inorganic phosphorus Electrolytes IgA	20 vs. 20
[16]	Diet	Col: 7 days Mat: 3 months	Term	Adiponectin Insulin	170 women
[17]	Diet Insulin	Col: 3 days Mat: 42–90 days	Term	Leptin insulin ghrelin adiponectin	48 vs. 48
[18]	Not evaluated	Col: 24–48 h Mat: 30 days	N/A	Leptin adiponectin insulin	12 vs. 21
[19]	Diet	Col/Mat	Term	Apelin nefastin-1 ghrelin	10 vs. 10
[20]	Diet	Col: 2 days Mat: 15 days	Term	Ghrelin	14 vs. 12 vs. 3 Pre-GDM
[21]	Not evaluated	Col: 72 h Mat: 6 weeks	Term	Irisin a SREBP-1c	33 vs. 33
[22]	Not evaluated	Col: –5 days Trans: 6–15 days Mat: >15 days	Term	Irisin Adropin Copeptin	15 vs. 15
[23]	Not evaluated	Col: 1 day Trans: 7 days Mat: 20 days	Term	Preptin Salusin Hepcidin	12 vs. 12
[24]	Not evaluated	Trans: 2 weeks	Term	Lactoferrin sIgA oligosaccharides	8 vs. 16
[25]	Diet	Col: 1–5 days Trans: 7–10 days Mat: 15–17 days	Term	Chemerin Dermcidin	26 vs. 27
[26]	Insulin	Col: First day of secretion and 2 days later	Term	Neutrophil gelatinase-associated lipocalin (NGAL) Complex NGAL/matrix metalloproteinase-9 (MMP-9)	13 vs. 22
[27]	Diet Insulin	Col: within 4 days Mat: 6 weeks	Term	Free amino acids	21 vs. 47
[28]	Diet Insulin Other medications	Col: 1–3 days	Term	Proteome	6 vs. 12
[29]	Not evaluated	Col:1–5 days	Term	Fatty acid composition	29 vs. 34
[30]	Diet insulin	Col: 1–3 day	Term	Sodium	17 vs. 116
[31]	Not evaluated	Col: 1 day	Term Preterm	Alpha-Tocopherol	20 vs. 31
[32]	Not evaluated	Col: 3–5 days Mat: 3 months	Term	MicroRNAs	19 vs. 47
[33]	Not evaluated	Col: 1–3 days Trans: 7–10 days Mat: 4 weeks	Term	Metabolome	90 vs. 94

**Table 2:** Results of the different studies included in the survey regarding GDM and HM.

Components	Effects	Reference
Energy content	Increase Decrease	Col/Trans/Mat: [13] Mat: [14]
Total protein content	No differences	Col: [13,14,15]
		Trans/mat: [14]
	Decrease	Trans/mat: [13]
Insulin	No differences	Col: [16,18] Mat: [18]
	Increase	Col: [17] Mat: [16,17]
Adiponectin	No differences Decrease	Col/Mat: [16,18] Col/Mat: [17]
Ghrelin	No differences Decrease	Trans: [17,19,20] Col/Mat: [17,19,20]
Irisin	Decrease	Col/Trans: [21,22]
		Mat: [21]
	No Differences	Mat: [22]
Apelins and Nesfatin-1	Increase	Col/Mat: [19]
Copeptin, adropin	No Differences	Col/Trans/Mat: [22]
Preptin	Increase No Differences	Col: [23] Trans/Mat: [23]
Salusin-alpha/-beta	Decrease No Differences	Col: [23] Trans/Mat: [23]
Pro-hepcidin and hepcidin-25	Increase No Differences	Col/Trans: [23] Mat: [23]
SREBP1-c	No Differences	Col/Mat: [21]
IgA	No Differences Decrease	Col: [15] Trans: [24]
Lactoferrin	Increase	Trans: [24]
Chemerin and Dermicin	Increase	Col/Trans/Mat: [21]
NGAL NGAL/MMP-9	No differences Increase	Col: [26] Col: [26]
Total saccharides content	No differences	Col/Trans: [13,14] Mat: [13]
	Decrease	Mat: [14]
Glucose	No differences	Col: [15]
Lactose	Decrease	Col: [15]
Oligosaccharides	No differences	Trans: [24]
Total lipid content	No differences	Col: [13,14] Trans: [13,14] Mat: [13]
	Decrease	Col: [15] Mat: [14]
Cholesterols	No differences	Col: [15]
triglycerides	No differences	Col: [15]
Fatty acid composition -γ-linolenic, eicosatrienoic, arachidonic, and docosatetraenoic	No differences Increase	Col: [29] Col: [29]
Potassium, Phosphorus and Calcium	No differences	Col: [15]
Sodium	No differences	Col: [15,30]
Vitamin E	No differences	Col: [31]
Micro RNA	No differences	Col/Mat: [32]

**Table 3:** Materials and Methods of the different studies included in the survey regarding insulin-dependent diabetes mellitus (IDDM) and HM.

Study	Metabolic Control in Pregnancy	Sample Time	Gestational Age	Parameters Analyzed	Sample Size IDDM vs. Control Women
[34]	Moderately controlled	24 h pool of mature milk: 16–90 days	Term	Total nitrogen, lactose, fat, trace minerals, lactoferrin, sIgA, glucose, sodium.	5 vs. 42
[35]	Poorly controlled	3, 4, 5, 6, 7 days	Term	Lipids, glucose, lactose, citrate, sodium, potassium, chloride, calcium, magnesium, total protein	1 vs. 13
[36]	Tightly controlled with continuous subcutaneous insulin infusion	0, 3, 5, 7, 9, 10, 12, 15, 17, 21, 25, 29, 35 days	Term	Triglycerides, lactose, protein, cholesterol, glucose, myoinositol, fatty acid	6 vs. 5
[37]	Tightly controlled	2, 3, 7, 14, 42, 84 days	Term Preterm	Prolactin	33 vs. 33 and 11 healthy reference women
[38]	Tightly controlled	3, 7, 14, 42, 84 days	Term Preterm	Total lipid, medium-chain fatty acids, saturated and monounsaturated long-chain fatty acid, long chain polyunsaturated fatty acid	33 vs. 33 and 11 healthy reference women
[39]	Tightly controlled	7, 14, 42, 84 days	Term Preterm	Vitamin E	33 vs. 33 and 11 healthy reference women
[40]	Not indicated	8–10 days	Term Preterm	Lactose, citrate and glucose	6 vs. 38
[41]	Tightly controlled	2, 3, 7, 14, 42, 84 days	Term Preterm	Lactose, total nitrogen, conductivity, osmolality	33 vs. 33 and 11 healthy reference women

**Table 4:** Results of the different studies included in the survey regarding IDDM and HM.

Components	Effects	Reference
Total protein content	No differences	Col/Trans/mat: [34,35,36]
Lactoferrin	No differences	Mat: [34]
Glucose	No differences Increase	Col/Trans/Mat: [36] Mat: [34]
Lactose	No differences	Col/Trans/Mat: [34,35,36]
Myoinositol	No differences	Col/Trans/Mat: [36]
Total lipid content	No differences Decrease	Col/Trans/Mat: [34,36,38] Col: [35]
Cholesterols	No differences Decrease	Col/Trans/Mat: [36] Col/Trans/Mat: [36]
Triglycerides	No differences	Col: [36]
Fatty Acid Profile	No differences	Col/Trans/Mat: [36]
medium-chain fatty acid	Decrease	Col: [34]
	No differences	Col/Trans/Mat: [38]
polyunsaturated fatty acids	Increase	Col: [35]
	Decrease	Col/Trans/Mat: [38]
oleic acid	Increase	Col: [35]
Potassium, magnesium and calcium	No differences	Col/Mat: [34,35]
Sodium	No differences Increase	Col: [35] Mat: [34]
Phosphorus, zinc, copper, iron	No differences	Mat: [34]
Chlorite and citrate	No differences	Col: [35]
Vitamin E	No differences	Col/Trans/Mat: [39]

## Abbreviations

American Academy of Pediatrics	AAP
Body mass index	BMI
Colostrum	Col
Free amino acids	FAAs
Gestational age	GA
Gestational diabetes mellitus	GDM
Human milk	HM
Insulin-dependent diabetes mellitus	IDDM
Mature milk	Mat
Matrix metalloproteinase-9	MMP-9
Neutrophil gelatinase-associated lipocalin	NGAL
Not applicable	N/A
Transitional milk	Trans
